# Weight bias and health care utilization: a scoping review

**DOI:** 10.1017/S1463423619000227

**Published:** 2019-07-22

**Authors:** Angela S. Alberga, Iyoma Y. Edache, Mary Forhan, Shelly Russell-Mayhew

**Affiliations:** 1 Department of Health, Kinesiology & Applied Physiology, Concordia University, Montreal, QC, Canada; 2 Department of Occupational Therapy, Faculty of Rehabilitation Medicine, University of Alberta, Edmonton, AB, Canada; 3 Werklund School of Education, University of Calgary, Calgary, AB, Canada

**Keywords:** obesity, primary health care, weight stigma

## Abstract

**Aim:**

The purpose of this scoping review was to explore the evidence on how perceptions and/or experiences of weight bias in primary health care influence engagement with and utilization of health care services by individuals with obesity.

**Background:**

Prior studies have found discrepancies in the use of health care services by individuals living with obesity; a greater body mass index has been associated with decreased health care utilization, and weight bias has been identified as a major barrier to engagement with health services.

**Methods:**

PubMed was searched from January 2000 to July 2017. Four reviewers independently selected 21 studies examining perceptions of weight bias and its impact on engagement with primary health care services.

**Findings:**

A thematic analysis was conducted on the 21 studies that were included in this scoping review. The following 10 themes were identified: contemptuous, patronizing, and disrespectful treatment, lack of training, ambivalence, attribution of all health issues to excess weight, assumptions about weight gain, barriers to health care utilization, expectation of differential health care treatment, low trust and poor communication, avoidance or delay of health services, and ‘doctor shopping’. Overall, our scoping review reveals how perceptions and/or experiences of weight bias from primary care health professionals negatively influence patient engagement with primary health care services.

## Introduction

Obesity management has been identified as a complex issue in primary health care (Brownell, 1982; Lyznicki *et al*., 2001). Discrepancies in the usage of health care services by individuals living with obesity have been reported in prior research (Drury and Louis, 2002; Coughlin *et al*., 2004; Ferrante *et al*., 2007; Aldrich and Hackley, 2010). In fact, it has been shown that having obesity impedes access to health care (Drury and Louis, 2002; Amy *et al*., 2006). Studies have documented a decrease in the use of health care services associated with an increasing body mass index (BMI) (Olson *et al*., 1994; Fontaine *et al*., 1998; Amy *et al*., 2006; Aldrich and Hackley, 2010). This includes reduced rates of routine breast and gynecological cancer screening tests among individuals with obesity compared to individuals with a BMI classified as normal (Adams *et al*., 1993; Fontaine *et al*., 1998; Aldrich and Hackley, 2010). When individuals with obesity avoid or delay health care services, the development of obesity-related comorbidities may go unnoticed, progress in severity, and become more difficult to treat. In this way, the avoidance of health care services could have detrimental implications for the prevention and management of obesity, its possible comorbidities, and other diseases (Phelan *et al*., 2015).

Weight bias and stigma, known as negative, prejudicial, or stereotypical beliefs and attitudes toward individuals based on their size, has been identified as a barrier to seeking health care services (Drury and Louis, 2002; Puhl and Heuer, 2009; Washington, 2011). Weight bias was cited as the fourth most common form of discrimination among US adults (Puhl *et al*., 2008). Over the past decade, the prevalence of weight bias has increased in the United States by 66% and has been documented in employment, education, and health care settings (Andreyeva *et al*., 2008; Puhl and Heuer, 2009). It has been reported that health professionals, specifically health care specialists in obesity treatment, hold strong implicit negative attitudes about individuals living with obesity (Teachman and Brownell, 2001). These stigmatizing attitudes are perceived and received by individuals with obesity and may contribute to the creation of multiple barriers to health care utilization (Drury and Louis, 2002).

Not only does weight bias pose adverse mental and physical health consequences such as exercise avoidance (Vartanian and Shaprow, 2008), anxiety (Hilbert *et al*., 2014), low self-esteem (Hilbert *et al*., 2014), and depression (Hilbert *et al*., 2014), but it also negatively impacts health care treatment outcomes (Carels *et al*., 2009). For example, a study compared people with severe obesity who experienced weight bias and those with severe obesity who did not experience weight bias. Those who experienced weight bias had a 1.5 kg/m^2^ greater BMI compared to those who did not report weight bias (Hansson and Rasmussen, 2014). In another study, participants who associated their obesity with more negative traits (higher weight bias) were more likely to drop out of an 18-week behavioral weight loss program compared to participants who evidenced lower levels of weight bias (Carels *et al*., 2009). These studies suggest that the stigma experienced by individuals with obesity may impede the adoptions and maintenance of healthy behaviors.

The purpose of this scoping review was to examine how perceptions and experiences of weight bias in individuals with obesity influence engagement in primary health care. As this is an emerging area of research, we used a scoping review methodology to provide a broad overview of the state of the evidence and to determine the value of undertaking a full systematic review. Note that for the purpose of this paper, ‘engagement in primary health care’ is defined as health care utilization, willingness to participate and be involved in health care visits (i.e., screening, prevention, regular checkups). Unless otherwise specified, the term ‘health professional’ is used in this paper to refer to nurses, physicians, and other allied health professionals (i.e., dietitians, health promotion specialists) working in a primary care setting.

## Methods

A scoping review of the literature was conducted using a predetermined specific research protocol based on the methodology described by Arksey and O’Malley (2005). Using this method, relevant literature is systematically identified, located, and summarized. This methodological approach is not intended to assess the quality of a study or provide quantitative synthesis of data. The purpose is to explore and chart the features of an emerging body of evidence and therefore is an effective approach to provide a broad overview of the literature and to identify research gaps. The methods we used to identify, select, and evaluate the evidence are described below. The Preferred Reporting Items for Systematic Review and Meta-Analyses extension for Scoping Reviews (PRISMA- ScR) was used to guide the reporting for this scoping review (Tricco *et al*., 2018).

## Literature search

A literature search was designed and conducted in consultation with an information specialist. In July 2017, we searched PubMed with a publications date limit between January 2000 to July 2017 and limited to English and French languages. Subject headings and key words were combined for concepts: weight bias and health care utilization. The keyword search strategy for each concept is presented in the Appendix. Additional articles not identified in the online database were either found as part of the researchers’ personal library or located from the reference lists of related articles.

## Study selection

**Figure 1.**
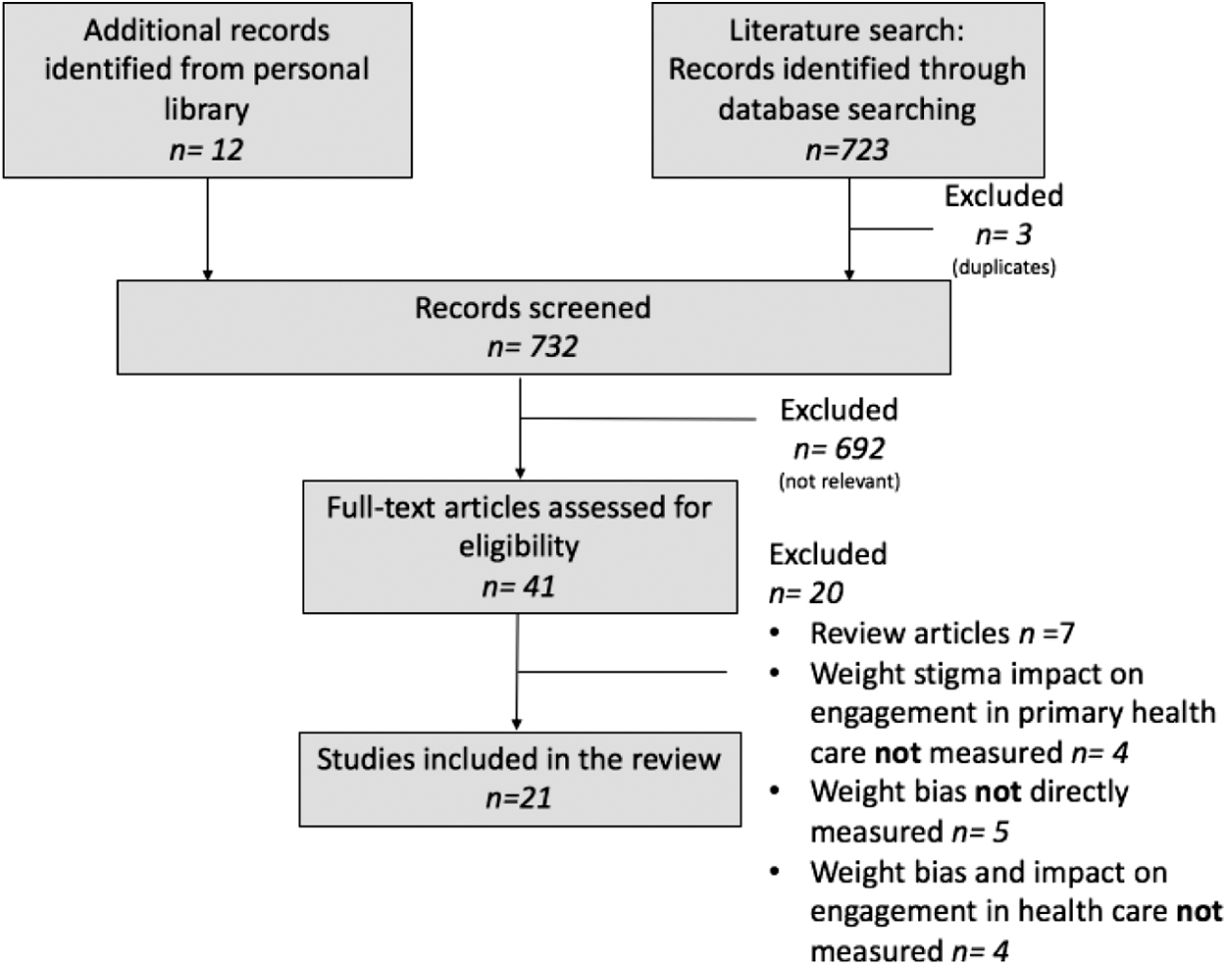
PRISMA-ScR flowchart illustrating the process of article selection.

Four independent reviewers screened titles and abstracts using the following keywords and their synonyms: weight bias, primary health care, and use of health care services. After screening by title and then by abstract, we assessed the remaining articles by reading the full text. Discrepancies were resolved by consensus between reviewers. Articles were included if they were original studies that examined the influence of perceived weight bias on engagement in primary health care, and described the stigma experienced by individuals with obesity in primary healthcare. We excluded articles that did not directly measure weight bias and/or engagement in primary health care and review papers on the topic. We made sure to include all original studies cited in review papers and omitted review papers to avoid duplication. We also included a PRISMA-SCR figure to detail the process and reasons for which studies were included and excluded (refer to Figure 1.)

## Data charting

Reviewers charted data for study characteristics (country, year of publication, study design, number of participants enrolled), patient population, and outcomes measured. All reviewers verified the data for accuracy and completeness. The data are presented in Table 1.

**Table 1.** Study characteristics

No.	Author	Year	Title	Geographical location	Study purpose	Primary health sector	Sample description	Sample BMI classification	Study design	Measures used	Main findings
1	Amy, N.K., Aalborg, A., Lyons, P., Keranen, L.	2006	Barriers to routine gynecological cancer screening for White and African-American obese women	California, USA	To investigate the factors that contribute to lower rates of gynecological cancer screening as related to women’s body size	Preventive cancer screening	Focus groups: *n* = 60 White and African American women 40–60 years old, *n* = 29 gynecological care providers (physician assistants, and nurse practitioners who provide gynecological care). Survey: *n* = 498 White and African American women 21–80 years, *n* = 129 health care providers	Women BMI: 25–35 kg/m^2^ (*n* = 131); >35–45 kg/m^2^ (*n* = 169); >45–55 kg/m^2^ (*n* = 121); >55 kg/m^2^ (*n* = 60)	Mixed methods	Focus group questions prompted discussions about perceptions and attitudes about gynecological cancer screening. Survey questions were based on focus group discussions. Women with obesity and health care providers were provided with different surveys.	Women reported weight-related barriers to health care access. These included disrespectful treatment, embarrassment at being weighed, negative attitudes, unsolicited advice about weight loss, and inappropriate medical equipment. With increases in BMI, a greater percentage of women reported delaying cancer screening tests.
2	Bottone, F.G., Musich, S., Wang, S.S., Hommer, C.E., Yeh, C.S., Hawkins, K.	2014	Obese older adults report high satisfaction and positive experiences with care	USA	To assess the impact of obesity on satisfaction and experiences with care in older adults	Did not exclusively examine one health sector (personal doctors and specialists)	N = 18,192 >65 years old with an AARP Medicare Supplement Insurance Plan insured by UnitedHealthcare Insurance Company in 10 states	Underweight (*n* = 516), normal (*n* = 7018), overweight (*n* = 6765) and obese (*n* = 3893)	Quantitative survey	Modified version of the Consumer Assessment of Healthcare Providers and Systems (CAHPS) survey mailed to the participants	Obesity was associated with higher patient satisfaction and better health care experiences. Patients with obesity had more doctor office visits about nutrition and exercise.
3	Brown, I., Thompson, J., Tod, A., Jones, G.	2006	Primary care support for tackling obesity: a qualitative study of the perceptions of obese patients	Sheffield, England	To explore obese person’s experiences and perceptions of support in primary care	General practice. Nurse practitioners or physicians	N = 28 (M = 10, F = 18) patients, >18 years from five general practice offices	Obese	Qualitative semi-structured interviews	Face-to-face 1-h interviews	Patients with obesity were ambivalent about accessing health services due to the lack of sensitive resources and ambiguous communication. Patients also perceived health professional ambivalence.
4	Buxton, B.K., Snethen, J.	2013	Obese women’s perceptions and experiences of healthcare and primary care providers: a phenomenological study	Pennsylvania, USA	To describe the experiences and perceptions of obese women with regard to stigma in health care	General practice. Nurse practitioners or physicians	N = 26 English-speaking women 27–66 years old	Obese	Phenomenological qualitative design using the Colaizzi method	Semi-structured, face-to-face 60–90 min interviews	All participants reported receiving some form of negative treatment from health care providers. Most participants did not report delaying or avoiding health care.
5	DeJoy, S.B., Bittner, K., Mandel, D.	2016	A qualitative study of the maternity care experiences of women with obesity: ‘more than just a number on the scale’	USA (13 different states)	To explore the experiences of women with obesity in the maternity care system in the United States	Maternity	N = 16 pregnant or recently postpartum women recruited from online communities for plus-size pregnant women	Obese	Qualitative interview	In-depth telephone interview ranging for 15 min–1 h	Most participants reported at least one stigmatizing maternity care experience. However, some participants did report being satisfied with the maternity services they received.
6	Drury, C.A.A., Louis, M.	2002	Exploring the association between body weight, stigma of obesity, and health care avoidance	Las Vegas, Nevada, USA	To explore the stigma of obesity and its effect on health care utilization	Did not exclusively examine one health sector (family practice, nurse practitioner, and gynecology)	N = 216 women from church sites 30–59 years old	Normal <27.5 kg/m^2^ (*n* = 137), mild obesity 27.5–30.0 kg/m^2^ (*n* = 19), moderate obesity >30–40 kg/m^2^ (*n* = 43), morbid obesity >40 kg/m^2^ (*n* = 11)	Quantitative survey	Questionnaire developed by Packer (1990) which included two questions from the Weight Locus of Control Scale modified by Packer, the Satisfaction with Medical Care Scale modified by Packer and the Rosenberg Self-Esteem Scale	Obesity stigma acts as a barrier to accessing health care. With increases in BMI, a greater number of participants delayed and/or avoided health care services.
7	Ferrante, J.M., Seaman, K., Bator, A., Ohman-Strickland, P., Gundersen, D., Clemow, L., Puhl, R.	2016	Impact of perceived weight stigma among underserved women on doctor-patient relationships	New Jersey, USA	To evaluate how perceptions of weight stigma among underserved women with obesity impacts doctor-patient relationships	General practice	N = 149 women 21–70 years old visiting physicians at four federally qualified health centers	Obese	Quantitative cross-sectional survey	The Stigma Situations in Health Care instrument and Consultation and Relational Empathy (CARE) measure	Increases in participant BMI classification was associated with increased likelihood of greater perceptions of weight stigma. With increases in stigma situations, there was a decrease in perceptions of physician empathy.
8	Forhan, M., Risdon, C., Solomon, P.	2013	Contributors to patient engagement in primary health care: perceptions of patients with obesity	Hamilton, Ontario, Canada	To identify issues associated with engagement in primary health care for patients with obesity	Family health team (family physicians, family medicine residents, and nurse practitioners)	N = 11(M = 2, F = 8) 19–64 years old registered with a primary care practice	Obese	Qualitative semi-structured interviews	Face-to-face and telephone interviews averaging 33 min	Feeling judged, lack of privacy, poor communication, and limited health provider knowledge about obesity were reported as barriers to primary health care engagement. Facilitators to engaging in primary health care included availability of resources, importance of relationship, and meaningful communication.
9	Gudzune, K.A., Bennett, W.L., Cooper, L.A., Bleich, S.N.	2014	Patients who feel judged about their weight have lower trust in their primary care providers	USA	To explore whether overweight and obese patients have less trust in their primary care providers (PCPs)	General practice	N = 600 (M = 312, F = 288) adults engaged in primary care in 2012	Overweight and obese	Quantitative cross-sectional survey	Survey questions assessed weight loss outcomes, doctor shopping behavior, and patient-provider relationship variables including duration, trust in PCP, and perceived weight judgment	21% of participants perceived weight related judgment from their PCPs. Participants who perceived judgment were less likely to trust their care provider.
10	Gudzune, K.A., Bennett, W.L., Cooper, L.A., Bleich, S.N.	2014	Perceived judgment about weight can negatively influence weight loss: a cross-sectional study of overweight and obese patients	USA	To examine the relationship between patient-perceived judgments about weight by primary care providers and self-reported weight loss	General practice	N = 600 (M = 312, F = 288) adults engaged in primary care in 2012	Overweight and obese	Quantitative cross-sectional survey	Survey questions assessed weight loss outcomes, doctor shopping behavior, and patient-provider relationship variables including duration, trust in PCP, and perceived weight judgment	Participants who perceived weight-related judgment from their primary care providers (21%) were more likely to attempt weight loss. However, perceptions of judgment were not associated with greater weight loss.
11	Gudzune, K.A., Bennett, W.L., Cooper, L.A., Clark, J.M., Bleich, S.N.	2014	Prior doctor shopping resulting from differential treatment correlated with differences in current patient-provider relationships	USA	To determine the prevalence of doctor shopping that is the result of differential treatment and to explore relationships between doctor shopping and current primary care relationships	General practice	N = 600 (M = 312, F = 288) adults engaged in primary care in 2012	Overweight and obese	Quantitative cross-sectional survey	Survey questions assessed weight loss outcomes, doctor shopping behavior, and patient-provider relationship variables including duration, trust in PCP, and perceived weight judgment	13% of participants reported previous doctor shopping behavior as a result of weight-based differential treatment. Doctor shopping behavior was associated with shorter durations of their current patient-provider relationships.
12	Gudzune, K.A., Beach, M.C., Roter, D.L., Cooper, L.A.	2013	Physicians build less rapport with obese patients	Baltimore, Maryland, USA	To describe the relationship between patient BMI and physician communication behaviors during a typical outpatient primary care visit	Routine follow-ups with primary care providers	N = 39 primary care physicians (PCPs) and *N* = 208 of their patients 18 years and older diagnosed with hypertension within 12 months of patient recruitment	Normal (*n* = 28), overweight (*n* = 60), and obese (*n* = 120)	Quantitative cross-sectional study	Audio-recorded outpatient encounters used to examine the frequency of communication behaviors in the patient-physician relationship	Primary care physicians engaged in less emotional rapport with patients with obesity or overweight, compared to normal weight patients.
13	Hansson, L.M., Rasmussen, F.	2014	Association between perceived health care stigmatization and BMI change	Sweden	To examine the association between experiences of health care stigmatization and BMI changes in men and women with normal weight and obesity	General practice	N = 2788 adults aged 25–64 years in 2008	Normal weight (*n* = 1064), moderate obesity (*n* = 1273), and severe obesity (*n* = 291) at the time of participation in the ULF survey	Quantitative survey	One question in the survey concerned perceived health care stigmatization. The Rosenberg’s Self-Esteem Scale and the Marlowe-Crowne social desirability scale	In the severe obesity group, health care stigmatization was associated with an increase in BMI by 1.5 kg/m^2^. With those classified as moderately obese, increases in BMI was associated with avoidance of health care and perceptions of insulting treatment.
14	Hilbert, A., Braehler, E., Haeuser, W., Zenger, M.	2014	Weight bias internalization, core self-evaluation, and health in overweight and obese persons	Germany	To examine a process model of self-stigma as well as the impact of core self-evaluation as a mediator between weight bias internalization, health outcomes, and health care utilization	Did not involve specific health care settings	N = 1158 (M = 629, F = 529) representative sample of German population 14–89 years old	Overweight (*n* = 931), obese (*n* = 227)	Quantitative survey	The Weight Bias Internalization Scale (WBIS), the Core Self-Evaluation Scale (CSES), the Patient health Questionnaire-2(PHQ-2), the Generalized Anxiety Disorder-2 (GAD-2), the Visual Analogue Scale (VAS) of health status, and the Health Care Utilization Questionnaire	In participants with overweight and obesity, lower core self-evaluation acts as a mediator in the relationship between weight bias internalization, health-related outcomes, and health care utilization.
15	Kaminsky, J., Gadaleta, D.	2002	A study of discrimination within the medical community as viewed by obese patients	Great Neck, New York, USA	To present the views and opinions of obesity surgery patients regarding care received before, during, and after weight loss surgery	Did not exclusively examine one health sector (primary care physicians and specialists)	N = 40 (M = 6, F = 34) obese adults 21–61 years old from four East Coast bariatric practices. Average preoperative weight of 145 kg	Obese	Quantitative survey	Survey assessing patient perceptions of physician and hospital staff attitudes, appropriateness of equipment, and level of care received from professional and non-professional medical personnel	17% of patients reported changing primary care physicians due to perceived physician indifference, lack of concern, or negative attitudes toward bariatric surgery.
16	Merill, E., Grassley, J.	2008	Women’s stories of their experiences as overweight patients	Texas, USA	To illuminate the meaning of women’s experiences as overweight patients in their encounters with health care services and health care providers	General practice and specialists	N = 8 women self-identified as being overweight patients. Ages 21–60 years old	Overweight and obese	Qualitative interviews. A hermeneutic phenomenological approach	In depth, face-to-face 50–90 min interviews. Participants were asked ‘Tell me a story, one you will never forget about going to your healthcare provider and your experience of being overweight’	Four major themes were identified: struggling to fit in, being dismissed, feeling not quite human, and refusing to give up.
17	Olson, C.L., Schumaker, H.D., Yawn, B.P.	1994	Overweight women delay medical care	La Crosse, Wisconsin, USA	To determine whether women delay or avoid health care because they are overweight	Community hospital	N = 310 female registered nurses (*n* = 225), licensed practical nurses (*n* = 26), nursing assistants (*n* = 13), health unit coordinators (*n* = 28), general psychiatric assistants (*n* = 1) and other (*n* = 17) 21–68 years old employed at St Francis Medical Center in July 1992	Underweight >20 kg/m^2^, normal weight 20–24.9 kg/m^2^, mild obesity 25–26.9 kg/m^2^ (*n* = 35), obese >27–34.9 kg/m^2^ (*n* = 75), very obese >35 kg/m^2^ (*n* = 11)	Quantitative survey	Visual analogue scale was used to assess perceptions of body weight. Survey questions assessed level of satisfaction with previous physician interactions concerning weight	BMI was positively associated with the delay of medical care. 12.7% of participants reported delaying or canceling a physician appointment due to weight concerns. Another small percentage (2.6) of participants reported keeping their appointments but refused to be weighed.
18	Pryor, W.	2002	The health care disadvantages of being obese	New South Wales, Australia	To describe the obese patients’ views about health care, myths and realities about obesity, and suggestions about how to improve health care for obese patients	General practice and specialists	A selection of messages posted by women with obesity on the *Big Beautiful Women Down Under* internet site	Obese	Informative bulletin	The *Big Beautiful Women Down Under* internet site	Health care professionals’ negative attitudes toward their patients with obesity are perceived by these patients. Inaccurate health professional assumptions about the eating habits and health behaviors of patients with obesity, inadequate equipment, and avoidance of general health care checkups were reported by women with obesity.
19	Puhl, R., Peterson, J.L., Luedicke, J.	2013	Motivating or stigmatizing? Public perceptions of weight-related language used by health providers	USA	To examine public preferences and perceptions of weight-based terminology	Routine checkup with a physician	N = 1064 (M = 417, F = 636) American adults 18–88 years old	Underweight (*n* = 47), normal (*n* = 351), overweight (*n* = 321), obese (*n* = 320)	Quantitative online survey	Likert scale (5 point) used to assess perceptions of 10 weight-related terms. Weight bias was assessed with the Fat Phobia Scale. Weight victimization was assessed with three forced choice questions (yes or no). Reactions to stigmatizing situations were assessed with a measure developed specifically for this study	Participants (19%) reported that they would avoid medical appointment if they felt stigmatized about their weight by their doctor. Participants (21%) also reported that they would seek a new doctor if they felt stigmatized about their weight by their doctor.
20	Russell, N., Carryer, J.	2013	Living large: the experiences of large-bodied women when accessing general practice services	New Zealand	To explore the experiences of large-bodied women (LBW) accessing general practice services	General practice	N = 8 self-identified LBW	Self-identified, large-bodied women (No BMI)	A qualitative descriptive inquiry that adopts a post-structural feminist lens during thematic analysis	Face-to-face interviews based on interview guide used in similar studies	Inappropriate humor, verbal insults, unmet health needs, and negative body language from health care providers were experiences of explicit negative weight bias reported by self-identified large bodied women.
21	Wadden, T.A., Anderson, D.A., Foster, G.D., Bennett, A., Steinberg, C., Sarwer, D.B.	2000	Obese women’s perceptions of their physicians’ weight management attitudes and practices	Philadelphia, Pennsylvania, USA	To examine obese women’s perceptions of their physicians’ weight management attitudes and practices	Weight management. (Physician, gynecologist, or nurse practitioner)	N = 259 women seeking treatment at one of three randomized control trials at the University of Pennsylvania with a history of weight loss and regain. Mean age of 44 ± 10 years	Obese	Quantitative questionnaire	A health care questionnaire developed by the authors measured patient satisfaction, frequency of physician discussions about weight, frequency of negative interactions with physicians about weight, and weight loss methods used by physicians. The Beck Depression inventory II was used to measure mood	Participants were less satisfied with the care they received for their obesity compared to the care they received for their general health. A small percentage of participants reported negative interactions with their physicians when weight management was discussed.

## Results

The literature search resulted in 720 unique articles. An additional 12 articles were identified from other sources resulting in a total of 732 articles. The 732 articles were screened and assessed for eligibility based on inclusion criteria. Of the 732 articles that we screened as potentially relevant, 21 studies met the inclusion criteria and were included in the review (Figure 1).

## Characteristics of included studies

Table 1 shows the characteristics of included studies. The majority of studies included in this review were carried out in the United States [*n* = 15 (71.4%)] and used quantitative methods [*n* = 13 (62%)]. Surveys were the most commonly used measure in quantitative studies [*n* = 13 (62%)]. The most commonly used qualitative method was interviews [*n* = 7 (33%)] including focus groups [*n* = 1 (4.8%)], telephone [*n* = 1 (4.8%)], face-to-face [*n* = 4 (19%)], or a combination of face-to-face and telephone [*n* = 1 (4.8%)].

The majority of the studies included mixed samples of both female and male participants [*n* = 11 (52.4%)]. The remaining 47.6% included only female participants (*n* = 10). Only participants with obesity were included in 38.1% (*n* = 8) of the studies. Other studies [*n* = 12 (57%)] compared different combinations of underweight, normal weight, overweight, and obese BMI classifications. One study did not measure participant BMI (4.8%).

Almost half of the studies [*n* = 9 (42.9%)] exclusively involved primary care physicians or nurse practitioners who work in general practice. These studies did not explicitly mention the types of primary health care services that the health professionals performed. Another 28.6% of studies did not exclusively examine one health sector (*n* = 6).

## Themes

The following 10 themes were identified after reviewing all articles: contemptuous, patronizing, and disrespectful treatment, lack of training, ambivalence, attribution of all health issues to excess weight, assumptions about weight gain, barriers to health care utilization, expectation of differential health care treatment, low trust and poor communication, avoidance or delay of health services, and ‘doctor shopping’. While reviewing the article summaries, the researchers compared the results of each article highlighting the emerging themes from the results. Next, relevant data from each study for a specific theme were sorted and charted together. The following section utilizes the data from the included studies to describe each theme.

### Contemptuous, patronizing, and disrespectful treatment

Four studies (Amy *et al*., 2006; Merrill and Grassley, 2008; Russell and Carryer, 2013; Buxton and Snethen, 2013) reported that participants with overweight and obesity experienced contemptuous, patronizing, and/or disrespectful treatment from health professionals. Contemptuous and patronizing behaviors involved verbal insults and inappropriate humor (Russell and Carryer, 2013). Participants with overweight and obesity reported feeling that they were being treated less respectfully than individuals classified as having a normal BMI (Amy *et al*., 2006). Participants perceived that weight-related advice from health professionals was delivered in a patronizing manner when health professionals insinuated that there was a simple solution to patients’ excess weight (Merrill and Grassley, 2008). Describing her experience, one woman stated:

*The doctor said, ‘Well, your blood pressure is high. You need to lose weight’. And I said, ‘I realize that’. He said, ‘Well, you just have to stop eating’. And I said, ‘If it would have been easy for me, I would have done it a long time ago…*

(Merrill and Grassley, 2008)

Buxton and Snethen also reported that patients with obesity received insensitive comments about their weight from their primary care practitioners (Buxton and Snethen, 2013). This was common when accessing emergency services where the patients had no established relationships with the primary care practitioner. One study that exclusively examined women with obesity reported that almost 80% of participants rarely or never had been treated disrespectfully (e.g., insulted or criticized for not trying hard enough) by their health professionals when discussing weight management (Wadden *et al*., 2000).

### Lack of training

Participants living with overweight and obesity perceived a lack of training among health professionals (Amy *et al*., 2006; Forhan *et al*., 2013; Russell and Carryer, 2013). Participants with obesity complained that health professionals involved in preventive screening and general practice did not demonstrate having knowledge about weight management and treatment services available for individuals living with obesity. Patients perceived the advice offered by their general practitioner as ineffective (Russell and Carryer, 2013). Amy *et al*. showed that over half of their sampled health professionals reported that they had no specific education on providing clinical gynecological examinations for patients with obesity (Amy *et al*., 2006).

### Ambivalence

Two studies (Brown *et al*., 2006; DeJoy *et al*., 2016) reported on patient ambivalence concerning the use of health services. Patients also perceived health professional ambivalence during weight-related health visits (Brown *et al*., 2006). In maternity care, women with obesity reported mixed feelings about whether or not to attend their antenatal and postpartum appointments as a result of the insensitive behavior they received from both past and current health professionals (DeJoy *et al*., 2016).

### Attribution of all health issues to excess weight

Patients with obesity complained of health professionals’ tendencies to attribute all of their other health issues to their excess weight (Amy *et al*., 2006; Brown *et al*., 2006; Merrill and Grassley, 2008; Forhan *et al*., 2013; Russell and Carryer, 2013; Ferrante *et al*., 2016). Patients felt that the emphasis health professionals put on their weight distracted from other health issues and resulted in feelings of not being listened to (Brown *et al*., 2006; Russell and Carryer, 2013). Attribution of all health issues to excess weight affected patients’ health utilization by increasing their reluctance to disclose the events surrounding the emergence of their symptoms, to see their general practitioner, or to express concern about a health issue (Brown *et al*., 2006). Patients wanted to avoid being weighed so as to keep the focus away from their weight and on the reasons why they visited their doctor (Forhan *et al*., 2013). Some participants (2.6%) reported attending their scheduled appointments but refused to be weighed (Olson *et al*., 1994). Collectively, the results of these studies were observed in preventive screening, in primary care services, and with general practitioners.

### Health professional assumptions about a patient’s weight gain

Patients indicated that health professionals often made assumptions about what it is like to live with obesity (Wadden *et al*., 2000; Pryor, 2002; Merrill and Grassley, 2008; Forhan *et al*., 2013; DeJoy *et al*., 2016; Ferrante *et al*., 2016). A participant in one study said:

*I guess I wonder if they may think why I don’t make the extra effort. That might be on the back of their head but they never actually say so. But, you get good at reading people when you are obese. You see it and you kind of know what they are thinking*.

(Forhan *et al*., 2013)

These assumptions were reported in both general practice and maternity care. Assumptions were made about how women’s weight gain occurred (e.g., being the result of lack of exercise and/or eating fast food and sweets) (DeJoy *et al*., 2016). One participant in this study said:

*They [health professionals] made judgments about what I ate, about how much I exercised. They never asked me; they just said things like ‘Don’t drink soda*,’ *which I don’t, and ‘Don’t eat candy bars*’*, which I don’t*.

(DeJoy *et al*., 2016)

These types of assumptions were often inaccurate, but health professionals did not listen when patients made efforts to correct them (Pryor, 2002; Merrill and Grassley, 2008; DeJoy *et al*., 2016). Wadden *et al*. showed that over 60% of patients complained that their physicians did not truly understand how difficult it was to be overweight (Wadden *et al*., 2000). In the same study, 24% of patients reported that their primary care practitioners sometimes did not believe them when they told them they do not eat that much.

### Barriers to health care utilization

Seven studies (Olson *et al*., 1994; Drury and Louis, 2002; Pryor, 2002; Amy *et al*., 2006; Forhan *et al*., 2013; Russell and Carryer, 2013; Ferrante *et al*., 2016) cited reasons for avoidance, delay, or cancellation of health care services observed with individuals with overweight or obesity. Barriers to health care utilization included unsolicited lecturing about weight loss (Olson *et al*., 1994; Wadden *et al*., 2000; Drury and Louis, 2002; Pryor, 2002; Amy *et al*., 2006; Ferrante *et al*., 2016); not wanting to get weighed (Olson *et al*., 1994; Drury and Louis, 2002); feeling embarrassed about their weight (Amy *et al*., 2006; Forhan *et al*., 2013); a fear of exposing their bodies (Russell and Carryer, 2013); undressing in health professionals’ offices (Drury and Louis, 2002); and inadequate hospital equipment such as small gowns, examination tables, chairs, and blood pressure cuffs (Pryor, 2002; Kaminsky and Gadaleta, 2002; Amy *et al*., 2006; Merrill and Grassley, 2008). A female participant expressed having to wait half an hour for a nurse to find an appropriately sized blood pressure cuff (Merrill and Grassley, 2008).

### Expectation of differential health care

Patients with obesity expected to receive different health care treatments because of their weight (Brown *et al*., 2006; DeJoy *et al*., 2016). Patient perceptions of weight bias resulted in the development of expectations of negative stereotypes in both social interactions and, to a lesser extent, health services (Brown *et al*., 2006). This was observed both during general practitioner visits and during maternity appointments. A study that exclusively involved pregnant or postpartum women with obesity reported that most participants expected differential maternity care due to their weight (DeJoy *et al*., 2016). Two-thirds of the participants reported at least one negative maternity care experience with health professionals when their weight was the focus of the interaction. Participants were suspicious that the care they received was a result of their size. The participants in this study perceived an increased medicalization of their pregnancy. Contrary to these results, a qualitative study conducted with women with obesity in a general practice setting reported that many participants denied being treated differently because of their weight and did not believe that they received less care (Buxton and Snethen, 2013).

### Low trust and poor communication

Several studies investigated the influence of weight bias on communication and level of trust in the patient–health professional relationship (Brown *et al*., 2006; Forhan *et al*., 2013; Russell and Carryer, 2013; Gudzune *et al*., 2013; 2014a). Patients were reluctant to initiate and express concerns about their weight to their health professionals (Brown *et al*., 2006). In this same study, patients reported not getting full explanations of why their weight was being raised by the health care professional as an issue for discussion. A small percentage of participants (10.9%) reported that they usually felt that they could not speak freely with doctors about their weight (Wadden *et al*., 2000). Patient awareness of their general practitioner’s negative preconceived notions limited the amount of information they were willing to share (Forhan *et al*., 2013). Patients with overweight and obesity who felt their primary care providers judged their weight were less likely to report high trust in these primary care practitioners (Gudzune *et al*., 2014a). Patients undergoing preventive screening were also dissatisfied with the insensitive and rushed communication from health professionals (Brown *et al*., 2006). During physician visits, primary care providers demonstrated lower levels of emotional rapport with patients with obesity and overweight compared to normal weight patients (Gudzune *et al*., 2013). On the contrary, a study, which asked participants to rate on a scale of 0–10 their level of trust in their current primary care practitioner, indicated that 74% of patients with overweight and obesity reported a high level of trust (scores ≥ 8) in their primary care practitioner. This high level of trust occurred regardless of whether or not participants had taken part in prior ‘doctor shopping’ (Gudzune *et al*., 2014b).

### ‘Doctor shopping’ as a result of the differential health care treatment

Studies have introduced the notion ‘doctor shopping’ as a consequence of experiencing weight bias in health care (Kaminsky and Gadaleta, 2002; Puhl *et al*., 2013; Gudzune *et al*., 2014b). If general practitioners did not provide the quality of care that the patients sought, they often searched for other health professionals who were better able to work with patients with obesity. In one study, 21% of participants reported that they would look for a new doctor if they perceived stigmatization about their weight (Puhl *et al*., 2013). Another study reported that 17% of participants changed primary care physicians due to physician indifference and negative attitudes toward bariatric surgery (Kaminsky and Gadaleta, 2002). Gudzune *et al*. reported that 13% of participants with overweight and obesity had cited previous doctor shopping as a result of differential treatment (Gudzune *et al*., 2014b).

### Avoidance or delay of health services

Seven studies found that weight bias among health professionals was associated with patient avoidance or delay of preventive screening, maternity, and general practitioner healthcare services (Olson *et al*., 1994; Drury and Louis, 2002; Pryor, 2002; Amy *et al*., 2006; Russell and Carryer, 2013; Puhl *et al*., 2013; Hansson and Rasmussen, 2014). Olson *et al*. reported that 32% of women with obesity and 55% of women with severe obesity reported delaying or canceling health care appointments because they knew they would have to be weighed during the appointment (Olson *et al*., 1994). Similarly, Russell and Carryer found that the majority of self-identified large-bodied women (BMI not reported) admitted to delaying and avoiding pelvic and breast examinations due to fears of judgment when exposing their bodies (Russell and Carryer, 2013). In terms of routine checkups, Puhl *et al*. reported that 19% of participants stated that they would avoid medical appointments if they perceived weight stigma (Puhl *et al*., 2013). Although seven studies reported the association between weight bias and decreased health care utilization, four studies reported different findings (Merrill and Grassley, 2008; Buxton and Snethen, 2013; Hilbert *et al*., 2014; Bottone *et al*., 2014). Buxton and Snethen reported that the majority of participants with obesity did not delay nor avoid health care (Buxton and Snethen, 2013). Further, Bottone *et al*. reported that 29.6% of patients with obesity reported visiting with their primary care provider three or more times in the past six months compared to 23.4% of patients with normal weight (Bottone *et al*., 2014). Hilbert *et al*. reported that a greater BMI predicted greater weight bias internalization and greater health care utilization (Hilbert *et al*., 2014). However, this study exclusively examined the influence of weight bias internalization on health care utilization. The theme ‘refusing to give up’ was highlighted in a study that reported on the experiences of patients classified as overweight in their encounter with health care professionals (Merrill and Grassley, 2008). ‘Refusing to give up’ illustrates the persistence of individuals with obesity to continue to try to control or lose weight. A female participant expressed that she would continue to pursue help from her physician:

*I was in her office a month ago and I said, ‘I want gastric bypass’. And she said, ‘Okay’. I said, ‘What?’ And she goes, ‘Okay’. I said, ‘You’re not going to argue with me about this and tell me to go eat less and exercise?’ And she said, ‘No’. And that was it*.

(Merrill and Grassley, 2008)

## Discussion

In this scoping review, we reviewed 21 published studies to examine the influence of weight bias on engagement in primary health care. We have highlighted the themes that emerged from an examination of these studies. In this section, we highlight inconsistencies, make recommendations for future research, and outline the strengths and limitations of this scoping review.

### Inconsistencies

The results of this review indicate that patients with overweight and obesity delay or avoid health care services as a result of health professionals’ weight bias. Receiving unsolicited lecturing about weight loss (Olson *et al*., 1994; Drury and Louis, 2002; Pryor, 2002; Amy *et al*., 2006; Ferrante *et al*., 2016), not wanting to get weighed (Olson *et al*., 1994; Drury and Louis, 2002), feeling embarrassed about their weight (Amy *et al*., 2006; Forhan *et al*., 2013), fear of exposing their bodies (Russell and Carryer, 2013), and inadequate hospital equipment such as small gowns, examination tables, chairs, and blood pressure cuffs (Pryor, 2002; Amy *et al*., 2006) were reported by participants as reasons for avoiding health care.

On the contrary, four studies in this review did not report a decreased use of health care services (Merrill and Grassley, 2008; Buxton and Snethen, 2013; Hilbert *et al*., 2014; Bottone *et al*., 2014). Hilbert *et al*. reported that a greater BMI predicted greater weight bias internalization known as greater health care utilization (Hilbert *et al*., 2014). However, this study exclusively examined a specific type of weight bias called weight bias internalization. Buxton and Snethen reported that the majority of participants with obesity did *not* delay nor avoid health care (Buxton and Snethen, 2013). Bottone *et al*. also reported that individuals with obesity were more likely to use more health care services (have three or more visits with their personal doctor in the past 6 months) (Bottone *et al*., 2014).

We speculate that these inconsistencies can be attributed to the fact that perceptions of weight bias in primary health care could differ depending on the sample being examined. For example, females might have different perceptions of weight bias compared to their male counterparts, and this might influence their engagement in primary health care services. Such inconsistencies in research examining the relationship between weight bias and health care utilization indicates that further study is warranted. Future studies should examine how weight bias influences the number of health care visits and should compare between sexes and ages. In addition, future studies should examine exclusively the different types of weight bias (explicit, implicit, and internalized) and the impact each type may have on health care utilization.

### Future research and recommendations

For improvements in patient engagement in the primary health care to occur, health professionals must first become aware of their weight bias attitudes and beliefs that could impact patient engagement in primary health care. It is only through awareness of one’s biases that conscious efforts can be made to impede their influence on behavior. Weight bias reduction interventions that promote discourse and positive interactions between patients with obesity and health professionals are recommended to improve patient and health provider communication (Alberga *et al*., 2016b) and mitigate the issue of differential perceptions of weight bias. Future research is needed to examine the effects of robust weight bias reduction interventions among pre-service and practicing health professionals.

The provision of health care equipment that is adequate and appropriate for all body types has the potential to influence health care utilization by individuals with obesity. Participants in four studies cited inadequate or inappropriately sized equipment as a barrier to health care utilization (Pryor, 2002; Kaminsky and Gadaleta, 2002; Amy *et al*., 2006; Merrill and Grassley, 2008). Addressing this barrier to health care utilization may result in patients feeling less embarrassed about attention being drawn to their body size due to inappropriate medical equipment.

There is a major gap in health professional training programs on obesity and weight bias (Amy *et al*., 2006; Forhan *et al*., 2013; Russell and Carryer, 2013). The need for educational programs aimed to improve knowledge of weight management and weight bias in primary health care has been identified by patients living with obesity (Amy *et al*., 2006; Forhan *et al*., 2013; Russell and Carryer, 2013). Improved training not only refers to providing educational information on the complexity of weight and the physiological aspects of obesity but also improving clinical skills to conduct sensitive and unbiased measurements of preventive screening tests or other health services. Such interventions could improve the effectiveness of treatment plans prescribed for patients with obesity and reduce ambivalence about obesity among patients and their health professionals. Avoidance or ambiguity of discussing weight is not an effective strategy to avoid weight stigmatization. Obesity Canada’s 5As of obesity management (Ask, Assess, Advise, Agree, Assist) are recommended for health practitioners usage in primary care to maintain sensitive, respectful, and non-judgmental conversations about weight management with people living with obesity (Rueda-Clausen *et al*., 2014).

More research is needed to fully examine the effects of weight bias in primary health care and on patient engagement in health care before a systematic review can be performed. As illustrated in this scoping review, many of the studies utilized a quantitative study design such as surveys. More qualitative research such as interviews and focus groups that examine patients’ perceptions and experiences of weight bias in primary health care are needed. Qualitative research and the lived experience of weight bias was identified as a strategic research priority among stakeholders in the field of obesity (Alberga *et al*., 2016a). In addition, this scoping review highlighted the lack of literature that exclusively examined the effects of health professional weight bias on men’s engagement in health care. More research on sex differences in health care engagement is needed before a systematic review may be performed.

### Strengths and limitations

The present study is the first, to our knowledge, that summarizes the existing literature on weight bias and patient engagement in primary health care. This scoping review provides a comprehensive summary of the results of the different studies that explored this topic. However, because our scoping review focused primarily on weight bias in primary care health professionals, conclusions drawn from this scoping review can only be made about primary care health professionals. We included three papers in this scoping review that reported three different outcomes albeit from the same sample of participants, which may be viewed as a limitation. Future research is warranted to examine the influence of weight bias on engagement in other health sectors and settings (e.g., diet and fitness industry, public health).

## Conclusion

This scoping review first identified perceived weight bias in primary health care evidenced by health care providers’ contemptuous, patronizing, and disrespectful treatment, lack of training, ambivalence, attribution and assumptions about patients’ weight and health. Second, it is clear that weight bias negatively affects patients’ engagement in primary health care through their perceived barriers to health care utilization, expectations of differential health care treatment, low trust and poor communication, avoidance or delay of health services, and ‘doctor shopping’. Future research and advocacy initiatives are needed to reduce weight bias among health professionals and improve quality of care and engagement in primary health care among patients living with obesity.

## Appendix

**Search 2017**

**Table:**

Concept: Weight bias		
S1	‘Weight Bias’	antifat[tiab] OR ‘anti fat’[tiab] OR ‘fat phobia’[tiab] OR ‘fat phobic’[tiab]
S2	Weight	‘Body Mass Index’[Mesh] OR ‘Body Weight’[Mesh] OR ‘obesity’[MeSH Terms] OR ‘overweight’[MeSH Terms] OR obese[tiab] OR obesity[tiab] OR overweight[tiab] OR ‘over weight’[tiab] OR weight[tiab]
S3	Bias	‘Bias (Epidemiology)’[Mesh] OR ‘prejudice’[MeSH Terms] OR ‘Social Stigma’[Mesh] OR ‘stereotyping’[MeSH Terms] OR bias[tiab] OR biased[tiab] OR biases[tiab] OR discriminate[tiab] OR discriminates[tiab] OR discriminated[tiab] OR discrimination[tiab] OR prejudice[tiab] OR prejudiced[tiab] OR stereotype[tiab] OR stereotypes[tiab] OR stereotyped[tiab] OR stereotyping[tiab] OR stigma[tiab] OR stigmas[tiab] OR stigmatization[tiab] OR stigmatize[tiab] OR stigmatized[tiab] OR stigmatizes[tiab] OR stigmatizing[tiab] OR stigmatisation[tiab] OR stigmatise[tiab] OR stigmatised[tiab] OR stigmatises[tiab] OR stigmatising[tiab] OR empathy[tiab] OR trust[tiab] OR ‘Negative interaction’[tiab] OR ‘negative encounter’[tiab] OR ‘negative experience’[tiab] OR shame[tiab] OR shaming[tiab] OR shamed[tiab] OR ‘Attitude of Health Personnel’[Mesh] OR ‘Physician-Patient Relations’[Mesh] OR ‘Nurse-Patient Relations’[Mesh]
S4	‘Weight Bias’ OR (Weight AND Bias)	S1 OR (S2 AND S3)
Concept: Health care utilization		
S5	‘Healthcare utilization’	‘Health Resources/utilization‘ [Mesh] OR ‘Patient Acceptance of Health Care’[Mesh] OR ‘Primary Health Care/utilization’[Mesh] OR ‘treatment seeking’[tiab]
S6	Healthcare	‘health care’[tiab] OR ‘health service’[tiab] OR ‘health services’[tiab] OR ‘family doctor’[tiab] OR ‘family practitioner’[tiab] OR ‘general doctor’[tiab] OR ‘general doctors’[tiab] OR ‘general practitioner’[tiab] OR ‘general practitioners’[tiab] OR GP[tiab] OR GPs[tiab] OR ‘primary care’[tiab] OR ‘medical care’[tiab] OR ‘Physicians, Primary Care’[Mesh] OR ‘family physician’[tiab] OR ‘primary care physician’[tiab]
S7	Utilization	avoid[tiab] OR avoidance[tiab] OR avoids[tiab] OR avoided[tiab] OR avoiding[tiab] OR consume[tiab] OR consumed[tiab] OR consumer[tiab] OR consumes[tiab] OR consuming[tiab] OR consumption[tiab] OR seek[tiab] OR seeking[tiab] OR seeks[tiab] OR sought[tiab] OR use[tiab] OR used[tiab] OR using[tiab] OR utilisation[tiab] OR utilise[tiab] OR utilised[tiab] OR utilises[tiab] OR utilization[tiab] OR utilize[tiab] OR utilized[tiab] OR utilizes[tiab] OR visit[tiab] OR visits[tiab] OR visited[tiab] OR visiting[tiab] OR engaged[tiab] OR engagement[tiab]
S8	‘Healthcare utilization’ OR (healthcare AND utilization)	S5 OR (S6 AND S7)
Final search’ weight bias AND healthcare utilization		
S9	Non-research articles	‘comment’[Publication Type] OR ‘editorial’[Publication Type] OR ‘letter’[Publication Type]
S10	Final search	(S4 AND S8) NOT S9
Filter	Publication date	2000/01/01 to 2017/12/31
Filter	Language	English OR French
